# Influence of shell compositions of solution blown PVP/PCL core–shell fibers on drug release and cell growth†

**DOI:** 10.1039/c8ra05485a

**Published:** 2018-09-19

**Authors:** Seok Chan Park, Min Jung Kim, Kyoungju Choi, Jooyoun Kim, Seong-O Choi

**Affiliations:** Nanotechnology Innovation Center of Kansas State (NICKS), Kansas State University Manhattan KS USA sochoi@ksu.edu; Department of Anatomy and Physiology, College of Veterinary Medicine, Kansas State University Manhattan KS USA; Department of Textiles, Merchandising and Fashion Design, College of Human Ecology, Seoul National University Seoul Republic of Korea jkim256@snu.ac.kr; Research Institute of Human Ecology, Seoul National University Seoul Republic of Korea

## Abstract

Developing a facile means of controlling drug release is of utmost interest in drug delivery systems. In this study, core–shell structured nanofibers containing a water-soluble porogen were fabricated *via* solution blow spinning, to be used as drug-loaded bioactive tissue scaffolds. Hydrophilic polyvinylpyrrolidone (PVP) and hydrophobic poly(ε-caprolactone) (PCL) were chosen to produce the core and the shell compartments of the fiber, respectively. In the core, a hydrophilic sulforhodamine B (SRB) dye was loaded as a model drug. In the PCL shell, two kinds of PVP with different molecular weights (40 kDa and 1300 kDa) were added, and the influence of PVP leaching on the SRB release and cell growth was investigated. The monolithic PCL-shelled fibers displayed a sustained SRB release with a weak burst effect. The addition of PVP in the shell induced a phase separation, forming microscale PVP domains. The PVP domain, acting as a porogen, was leached out in the medium and, as a result, the burst release of SRB was promoted. This burst effect was more prominent with the lower molecular weight PVP. The biocompatibility of the core–shell fibers was evaluated with human epidermal keratinocytes (HEK) by a cell viability assay and microscopic observation of cell proliferation. The HEK cells on fibers with a PVP/PCL composite shell formed self-assembled spherical clusters, displaying higher cell viability and proliferation than those on the monolithic PCL-shelled fibers that induced HEK cell growth in two-dimensional monolayers. The results demonstrate that the presence of hydrophilic porogens on tissue scaffolds can accelerate drug release and enhance cell proliferation by increasing surface wettability, roughness and porosity. The findings of this study provide a basic insight into the construction of bioactive three-dimensional tissue scaffolds.

## Abstract 2

The presence of hydrophilic porogens on the surface of core–shell fibers can accelerate drug release and enhance cell proliferation.

## Graphical abstract

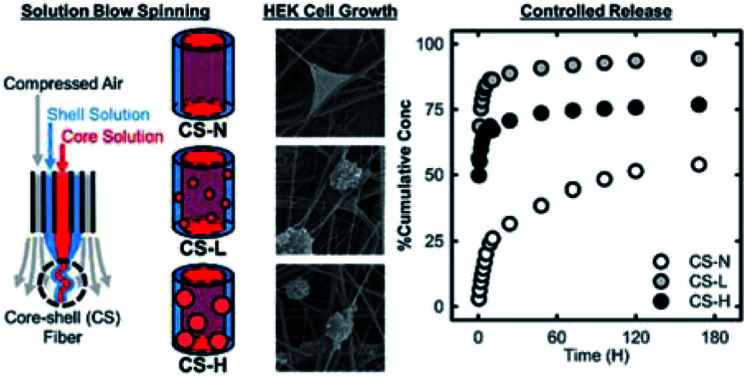

## Introduction

With the structural similarity of fibrous materials to the extracellular matrix (ECM), ultra-thin fibers have been used to reproduce the intricate network of ECM in target tissues such as cartilages, bones, arterial blood vessels and nerves.^1–6^ The scaffold comprising ultra-thin fibers affords highly porous microstructures with interconnected pores and large surface area, which are essential to resemble a native ECM. As the high specific surface area of fine fibers is desirable for an effective release of incorporated drugs,^7,8^ fibrous scaffolds containing bioactive molecules have been fabricated to enhance the therapeutic effect. For example, bioactive molecules embedded in a fibrous scaffold can be used as a guidance signal to induce specific cell behaviors, or as an anti-infective during tissue regeneration. With various bioactive functionalities, fibrous scaffolds have been applied to tissue engineering,^9,10^ wound healing,^11^ postoperative anti-adhesion,^12^ guided bone regeneration^13^ and *in vivo* local delivery.^14^

In drug delivery, controlled release of encapsulated bioactive substances is crucial to maximize the therapeutic efficiency and to minimize the side effects.^15–18^ It has been revealed that the physicochemical properties of a polymeric matrix and encapsulated agents are primary parameters governing the drug release behavior. For example, spontaneous drug release is influenced by degradability and wettability of drug-carrying vehicles, diffusivity of the guest molecules, and the compatibility of a drug in a polymeric matrix.^19–23^ Modification of structural parameters of drug delivery systems is another important means of manipulating release behavior. Among various approaches, constructing core–shell structured drug carriers, such as coaxial fibers or emulsions, holds great potential.^15,24–26^ By varying the material compositions or thickness of the shell and the core,^26,27^ the release profile can be fine-tuned over a wide range of time scales.

While electrospinning is the most popular method to fabricate fibrous drug carriers and scaffolds,^28–30^ the use of a high voltage may limit the application of bioactives such as highly charged drugs and live cells. Low production efficiency of the electrospinning process is another limitation for the large-scale manufacturing. In order to overcome these limitations, solution blow spinning (SBS) can be utilized as an alternative process to generate micro/nanofibers with higher productivity.^31^ SBS produces fibers from polymer solutions through a high-velocity gas flow that enables rapid elongation and solidification of the precursor solution. This process has been extensively used over the past few years as an efficient and economical way of scale-up production of micro-/nanofibers with a broad range of spinnable materials.^32^

This study aims to develop biocompatible drug-loaded core–shell fibers *via* solution blow spinning and explore the applicability of the solution blown fibers to the drug-carrying tissue scaffolds for regenerative therapy with controlled drug release. For fabricating core–shell fibers, a coaxial setup for solution blow spinning was used. Water-soluble polyvinylpyrrolidone (PVP) was used for constructing the fiber core in which a hydrophilic fluorescence dye, sulforhodamine B (SRB) was incorporated as a model drug. For the shell formation, biodegradable poly(ε-caprolactone) (PCL) was used with or without PVP emulsions, which act as pore-forming agents (porogens) on the PCL shell upon exposure to an aqueous medium. Two different molecular weights (40 and 1300 kDa) of PVP were used as porogens. The results demonstrated that the dissolution rate of PVP porogens in the fiber shell depended on the molecular weight of PVP, thus influencing the release rate of SRB. The cell culture study with human epidermal keratinocytes (HEK) suggested that the fiber mats containing PVP in the PCL shell would provide an environment suitable for three-dimensional cell growth.

## Experimental section

### Materials

Polyvinylpyrrolidone (PVP, MW 40 and 1300 kDa) and sulforhodamine B (SRB) were purchased from Sigma-Aldrich (St. Louis, MO, USA). Poly(ε-caprolactone) (PCL, MW 144.8 kDa, PDI 1.80) was purchased from Lactel Absorbable Polymers (Birmingham, AL, USA). Chloroform (certified ACS grade), *N*,*N*-dimethylformamide (DMF, certified ACS grade) and phosphate-buffered saline (PBS, Corning™ cellgro™ Cell Culture Phosphate Buffered Saline 1×) were purchased from Fisher Scientific (Waltham, MA, USA). Ethanol (EtOH, 200 proof, USP grade) was purchased from Decon™ Labs (King of Prussia, PA, USA).

### Solution blow spinning of PVP/PCL core–shell fiber

**Fig. 1:**
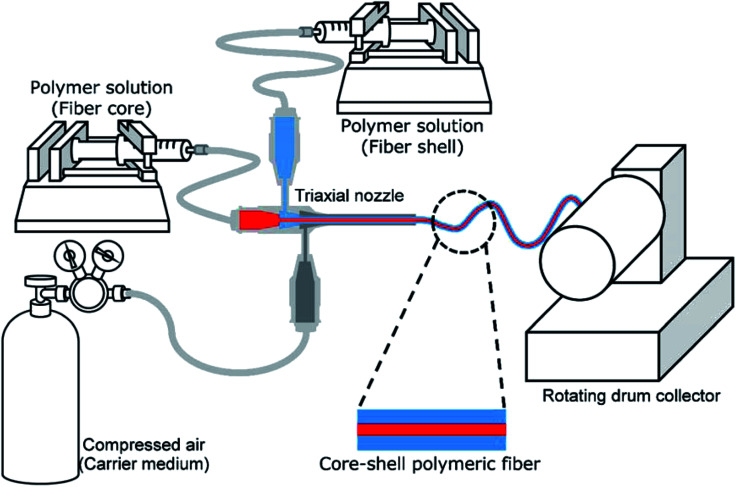
Schematic diagram of solution blow spinning setup for core–shell fiber formation.

Core–shell fibers containing a model drug within the core were fabricated *via* solution blow spinning (SBS). The experimental setup for the SBS process is illustrated in Fig. 1. A triaxial needle (Ramé-Hart Instrument Co., Succasunna, NJ, USA) was used as a spinneret, which is composed of 22 G (inner), 16 G (middle) and 12 G (outer) concentric needles. Polymer solutions for the core and shell were fed into the inner and middle needles, respectively, using syringe pumps (AL-1000, World Precision Instrument, Sarasota, FL, USA), and the outer needle was connected to a compressed air source. Fibers were collected on a rotating drum collector (150 rpm; NNC-DC90, NanoNC, Seoul, South Korea), which was placed 20 cm from the spinneret.

The PVP/PCL core–shell fibers with three different shell compositions were prepared: PCL only (sample code: CS-N), PCL with low molecular weight (40 kDa) PVP (sample code: CS-L) and PCL with high molecular weight (1300 kDa) PVP (sample code: CS-H). To prepare 0.5% (v/v) PVP/PCL blend for the shell, 50 μl of 20% (w/v) PVP solution in 1 : 1 water/ethanol (EtOH) was added dropwise to 10 ml of 10% (w/v) PCL solution in 7 : 1 chloroform/DMF and emulsified by ultrasonication for 3 min (Sonics VCX-130 Vibra-Cell Ultrasonic Liquid Processor, Sonics & Materials, Inc., Newtown, CT, USA). The fiber core was produced from 1300 kDa PVP (10% w/v in EtOH) doped with 0.25 mM SRB. The solution feed rates used in this study were 27.2 μl min^−1^ and 300 μl min^−1^ for the core and shell solutions, respectively. The gas pressure was 10 psi. The composition of solutions and fabrication parameters are summarized in Table 1.

**Table 1:** Solution compositions and SBS process parameters used for core–shell fiber fabrication

Sample code	Solution composition				Flow rate (μl min^−1^)		Air pressure (psi)
	Core		Shell				
	Model drug	Main polymer	Emulsion	Main polymer	Core	Shell	
CS-N	SRB 0.25 mM	PVP 1.3 MDa 10% (w/v) in EtOH	None	PCL 10% (w/v) in chloroform/DMF (7 : 1)	27.2	300	10
CS-L			PVP 40 kDa 20% (w/v) in water/EtOH (1 : 1)				
CS-H			PVP 1.3 MDa 20% (w/v) in water/EtOH (1 : 1)				

### Phase separation of the polymer blends in the fiber shell

To examine the phase separation of PCL and PVP in the pre-spinning solution, 10 μl of PVP/PCL blends used for the fiber fabrication was placed between two glass coverslips and was observed under an inverted fluorescence microscope (Olympus IX-73, Tokyo, Japan) coupled with a digital camera and integrated software. The existence of PVP in the PCL shell was observed by energy-dispersive X-ray spectroscopy (EDS; Hitachi S-3400N, Tokyo, Japan) with a working distance of 16 mm and an acceleration voltage of 25 kV. CS-L and CS-H were placed on a pin stub with conductive carbon tape, and were subjected to EDS mapping without metal coating.

### Characterization of SBS nanofibers by electron microscopy

To examine the morphology of core–shell fibers, the collected nonwoven fiber mats were sputter-coated with 10 nm-thick palladium and observed by a scanning electron microscope (SEM; Hitachi S-3400N, Tokyo, Japan) with a working distance of 10 mm and an acceleration voltage of 5 kV. Formation of core–shell structures was observed by transmission electron microscopy at 80 kV (TEM; FEI Tecnai F20 XT Field Emission TEM; Tecnai G2 Spirit BioTWIN TEM). TEM samples were prepared by directly collecting fibers onto TEM grids (Lacey carbon, 300 mesh, Ted Pella, Inc., Redding, CA, USA).

### Water contact angle measurement

Surface wettability of the core–shell fiber mats was examined by static water contact angle (WCA) measurement. A 4 μl distilled water drop was placed on the fiber mats, and WCA was measured by an optical tensiometer (Attension Theta, Biolin Scientific, Paramus, NJ, USA). Due to the fluffy surface of the fiber mats, the baseline of the substrate was carefully determined. Measurements were done at least five times at different locations from 2 samples of each fiber type.

### *In vitro* drug release study

The fiber mats were cut into strips (75–85 mg), and three strips were prepared from each fiber. Each sample was immersed in 5 ml of PBS at 32 °C. 1 ml of PBS was collected at predetermined time points to measure the amount of SRB released from each fiber sample, and the sample tubes were refilled with 1 ml of fresh PBS. The concentration of SRB was measured using a multi-mode microplate reader (Synergy H1 Hybrid, BioTek, Winooski, VT, USA) with an excitation wavelength of 555 nm and an emission wavelength of 585 nm. All measurements were done in triplicate from separately incubated samples (*i.e.*, total 9 measurements for each type of fiber), and the percent cumulative concentrations were calculated as a function of release time:
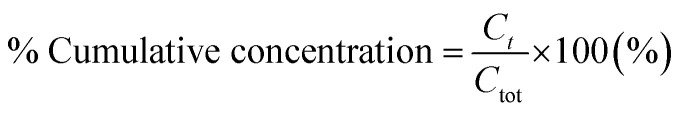
where *C*_*t*_ is the cumulative concentration of SRB released at time *t* and *C*_tot_ is the total concentration of SRB in the fiber mat.

To determine the total concentration *C*_tot_, SRB was extracted from the fiber by phase separation. For SRB extraction, a fiber sample with known weight (approximately 40 mg) was dissolved in 5 ml of chloroform, and the chloroformic solution was vigorously mixed with 5 ml of PBS to dissolve SRB in PBS. The mixture was then left in dark for 24 h for phase separation and was further subjected to centrifugation (4696 × *g*, 45 min) to complete the separation process. After phase separation, the supernatant PBS containing SRB was collected, and the concentration of SRB partitioned in the PBS phase was determined by fluorescence spectroscopy. SRB standard solutions were prepared in the same way to reflect the partition ratio of SRB between PBS and chloroform in the concentration calculation.

### Human epidermal keratinocytes cultures

Cryopreserved primary neonatal human epidermal keratinocytes (HEK) were obtained from Lonza(Walkersville, MD, USA). HEK cells were cultured in complete keratinocyte medium (KM) supplemented with keratinocyte growth supplement (ScienCell™ Research Laboratories, Carlsbad, CA, USA) and expanded in a poly-l-lysine-coated (2 μg cm^−2^, ScienCell™ Research Laboratories, Carlsbad, CA, USA) T-75 flask to approximately 80% confluency. The medium was replaced with fresh complete KM every 2 days. After trypsinization, 9.5 × 10^3^ cells in complete KM were seeded onto poly-l-lysine-coated fiber samples. Prior to poly-l-lysine coating, fiber mats were cut into a circular shape with a diameter of 10 mm using a biopsy punch. The morphological changes of fibers after poly-l-lysine coating was observed by SEM (Fig. S1†). To avoid deformation and folding during incubation, the fiber discs were placed in 24-well plates using a homemade polylactic acid (PLA) holder. HEK cells were grown at 37 °C in a humidified atmosphere of 95% air and 5% CO_2_, and cell morphology and proliferation were examined after 48 h incubation.

### Characterization of HEK morphology by microscopy

Morphology of HEK cells grown on the fiber mats was assessed by SEM and confocal fluorescence microscopy. For SEM analysis, samples incubated for 48 h were fixed with a mixture of 4% formaldehyde and 1% glutaraldehyde in phosphate buffer (Thermo Fisher Scientific, Waltham, MA, USA) at 4 °C overnight. After triplicate rinsing with 0.1 M phosphate buffer (pH 7.2–7.4) and double distilled water for 5 min, respectively, the samples were dehydrated in a graded ethanol series (70%, 80%, 95% and 100%, 30 min each). After incubation in 100% ethanol for 30 min (repeated 3 times), the samples were immersed into hexamethyldisilazane for 5 min (repeated twice) to complete dehydration. The samples were further dried under vacuum and sputter-coated with 10 nm-thick palladium for SEM imaging. The SEM imaging was performed with a working distance of 10 mm and an acceleration voltage of 5 kV.

For confocal fluorescence microscopy, HEK cells were double-stained with fluorescein diacetate (FDA; 0.5–5 mg ml^−1^, Sigma-Aldrich, St. Louis, MO, USA) for viable cells and propidium iodide (PI; 2 mg ml^−1^, Thermo Fisher Scientific, Waltham, MA, USA) for necrotic/dead cells. Prior to staining, cells were washed with PBS. After 5 min incubation in the dark, cells were washed with PBS and examined using a confocal microscope (LSM 700, Carl Zeiss Microscopy, Jena, Germany). Confocal images were recorded in two detection channels (560–800 nm for PI and 300–550 nm for FDA) with excitation at 555 nm and 488 nm, respectively.

### Measurement of HEK proliferation

HEK proliferation was quantitatively measured by the alamarBlue assay (Thermo Fisher Scientific, Waltham, MA, USA) as previously described.^33^ Briefly, HEK cells grown on the fiber mats for 48 h were washed with PBS and incubated with alamarBlue reagent in complete KM (1 : 10 volume ratio). After 3 h incubation at 37 °C, fluorescence was quantified using a multi-mode microplate reader (Synergy H1 Hybrid, BioTek, Winooski, VT, USA) at excitation/emission of 555/585 nm. All experiment was conducted in triplicates, and fluorescence intensities were compared.

### Statistics and data analysis

Data from WCA were compared using a Student's *t*-test with equal variances. A value of *p* < 0.05 was considered statistically significant. One-way analysis of variance (ANOVA) was conducted using GraphPad Prism 6 (GraphPad Software Inc., La Holla, CA, USA) to assess the effects of nanofiber mats on HEK proliferation. If significant, the multiple comparison was performed with Tukey's honest significant difference (HSD) test at a *p* < 0.05. All data were presented as mean ± standard deviation (S.D.).

## Results and discussion

### Formation of PVP/PCL core–shell fibers

Core–shell nanofibers were fabricated *via* solution blow spinning (SBS) using a triaxial needle. The SBS process is a relatively safe and convenient way of fabricating fibrous mats because it does not require electrostatic drive force nor conductive collector. Water-soluble PVP and a biodegradable polyester, PCL were used to form the core and the shell compartments, respectively. As airflow to the outer nozzle orifice was initiated, spinning solutions were drawn and formed fine fibers as a result of solvent evaporation and solidification of elongated polymer strands. The process parameters and solution compositions were carefully adjusted to produce continuous fibers, avoiding dripping or clotting of the polymer solutions at the exit orifice of the spinneret.

**Fig. 2:**
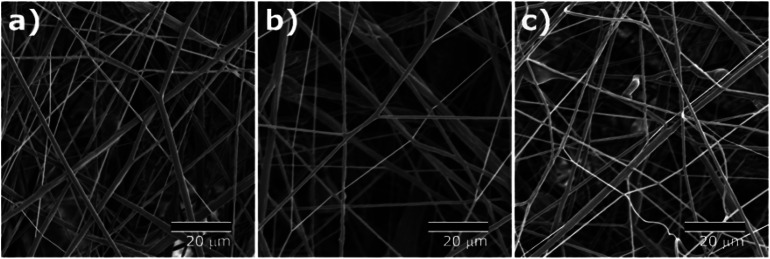
SEM images of solution blown fibers with (a) monolithic PCL shell (CS-N), (b) PCL shell with 40 kDa PVP (CS-L), and (c) PCL shell with 1300 kDa PVP (CS-H).

As shown in Fig. 2, nonwoven fiber mats were collected with a random orientation. SEM images of the fiber mats exhibit bead-free, smooth fibrous surfaces of all the core–shell fibers with three different shell formulations, and formation of porous scaffold comprising continuous fibers.

**Fig. 3:**
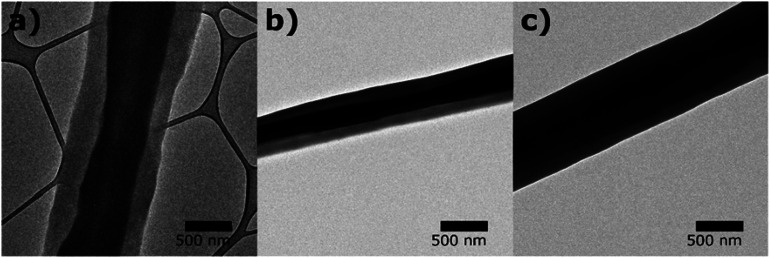
TEM images of core–shell structures of solution blown fibers with (a) monolithic PCL shell (CS-N), (b) PCL shell with 40 kDa PVP (CS-L), and (c) PCL shell with 1300 kDa PVP (CS-H).

TEM images (Fig. 3) show clear boundaries between the core (PVP) and the shell (PCL or PVP/PCL blends), confirming the core and shell construction of the fibers. While fiber diameters varied from 460 nm to 1050 nm, the diameter ratio of shell to core appeared consistent to be 2 : 1. The presence of a continuous core inside the solution blown fibers indicates the complete encapsulation of the core material and consequently, inhibiting the immediate release of molecules incorporated in the core. The morphologies of the fiber mats suggest their potential applications in controlled release scaffolds.

### Phase separation of polymer blends in fiber shell

**Fig. 4:**
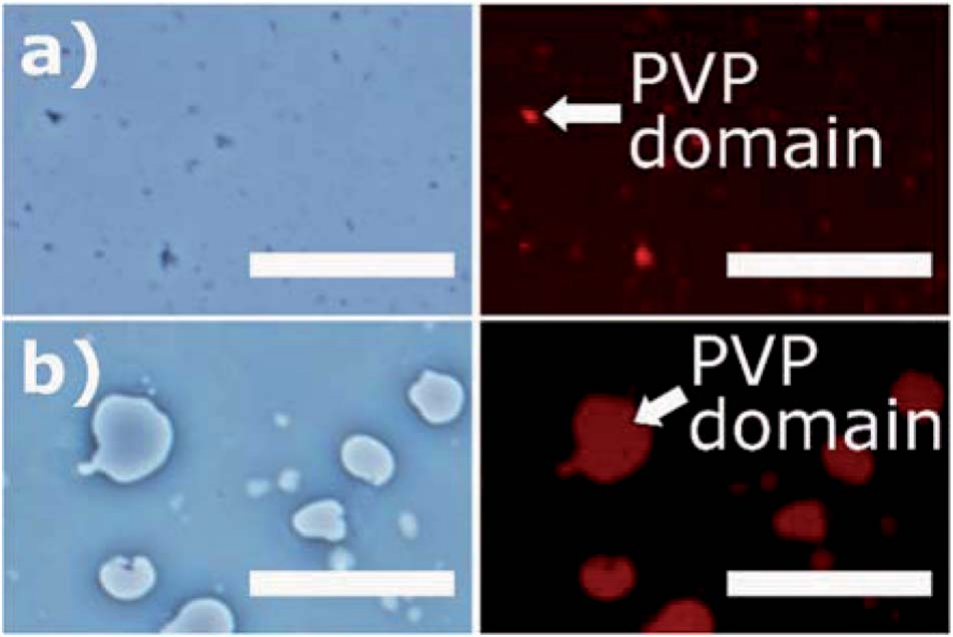
Bright-field and fluorescence microscopic images of PVP/PCL blends. (a) 40 kDa PVP and (b) 1300 kDa PVP in PCL. Red spots in the fluorescence images (right column) represent the distribution of SRB-doped PVP phase (scale bar: 50 μm).

In Fig. 4, the PVP/PCL solution mixture was observed under optical and fluorescence microscopy^25,34^ to monitor the phase separation of PVP and PCL domains in the solution mixture. For visualization, PVP emulsion was doped with SRB. The microscopic images shows a clear separation between PVP and PCL phases, indicating that two polymers are immiscible. The size of emulsion varied depending upon the molecular weight of PVP. The lower molecular weight PVP (40 kDa) produced uniform, small-sized (<5 μm) emulsions (Fig. 4(a)), while the higher molecular weight PVP (1300 kDa) produced uneven dispersion of larger emulsions (Fig. 4(b)).^35,36^ It should be noted that, as the solvent evaporates, the size of the PVP emulsion was shrunk, thereby forming submicron-sized PVP domains on the solidified fiber shell. While the PVP emulsions in the PVP/PCL were observed in the liquid phase, those in the solid phase were hardly observable.

Elemental mapping was performed to observe the shell compositions of CS-L and CS-H (Fig. S2†). As nitrogen exists only in PVP, the distribution of nitrogen should show the PVP-rich domains. Although phase separation between PCL-rich domain and PVP-rich domain was not clearly shown from this experiment, the EDS map indicates the presence of PVP in both CS-L and CS-H shells (Fig. S2c and d†).

The water-soluble PVP domains would act as a porogen, creating pores on the PVP-PCL shells by the selective dissolution of PVP in aqueous media. The dissolution process of the PVP domains and the resulting pore configuration would influence the diffusion of SRB encapsulated in the fiber core. In addition, distinct sub-micron topologies would be generated on the fiber surface depending on the dissolution rate and the molecular weight of PVP. The created surface topologies may also affect the growth and proliferation of the incubated cells. Though it was attempted to observe the pores in the fiber surface after immersion in PBS solution, the pores on fiber samples were not clearly observed due to the limited resolution of the imaging techniques.

### Characterization of wettability

**Fig. 5:**
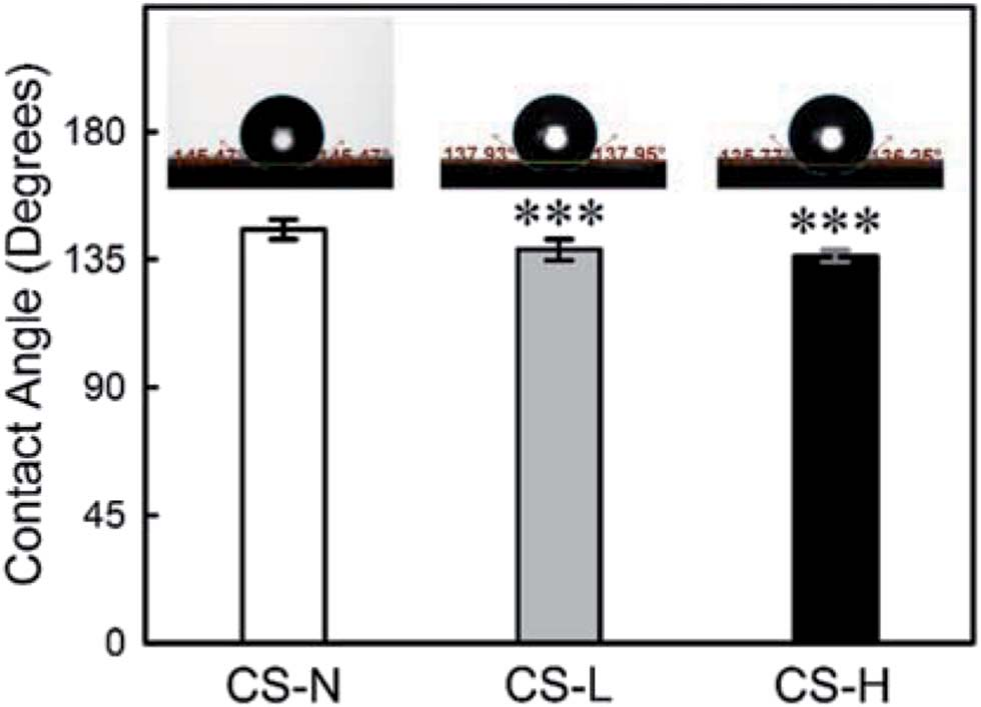
Water contact angle of core–shell fiber mats with different shell compositions. Left: monolithic PCL shell (CS-N), Middle: PCL shell with 40 kDa PVP (CS-L), Right: PCL shell with 1300 kDa PVP (CS-H). Inset images show a 4 μl water droplet on each fiber mat. Data represent mean ± S.D. ***p < 0.001.

The wettability of PVP/PCL core–shell fibers was characterized by measuring water contact angles (WCA) (Fig. 5) as the hydrophilicity/hydrophobicity of scaffolds influences the initial adhesion and further proliferation of cells.^37–39^ Also, the wettability of a material has a significant effect on the diffusion process of substances incorporated in the material.

When the shell is composed of PCL only, WCA was 145° ± 4° (*n* = 12) due to PCL's hydrophobic nature. The addition of PVP into the fiber shell reduced the WCA to 138° ± 4° (*n* = 12) for CS-L, and 136° ± 2° (*n* = 10) for CS-H, respectively. The increase in surface wettability, resulted from PVP blending in the shell, is expected to promote the mass transfer of incorporated molecules from the core.

### SRB dye release from core–shell fibers

**Fig. 6:**
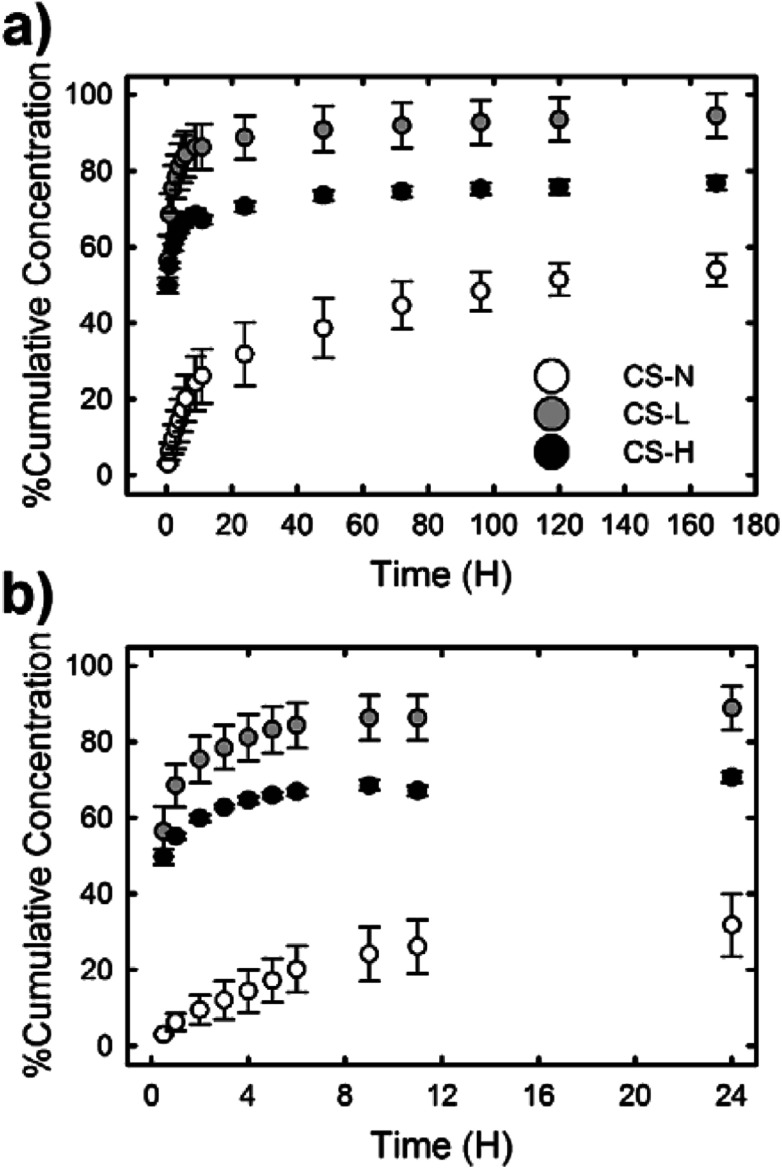
SRB dye release profiles of PVP/PCL core–shell fibers for (a) 7 days and (b) 24 h.

Fig. 6 shows the *in vitro* release profiles of SRB molecules incorporated in the PVP core of three different core–shell fibers. All samples exhibited an initial burst release followed by a slow release of SRB. The release rate and the amount of released dye over 7 days, however, varied depending on the composition of the fiber shell. The burst effect of each fiber during the first 24 h was distinct (Fig. 6(b)). The fiber containing low molecular weight PVP in the shell (CS-L) displayed the fastest release of SRB; ∼56% and ∼86% of the dye were released during the first 30 min and 24 h, respectively. After the initial burst, the release rate kept decreasing considerably and only an additional 5% of SRB was released between 24 h and 168 h. When high molecular weight PVP was added to the shell (CS-H), overall drug release including the initial burst was suppressed compared to CS-L, resulting in the cumulative release rates of approximately 50, 67 and 77% at 0.5 h, 12 h and 168 h, respectively. In contrast to the fibers with PVP/PCL shell, the fiber with monolithic PCL shell (CS-N) exhibited a significantly reduced initial burst (approximately 3% release during the first 30 min and less than 20% release within 4 h) due to the shielding effect by the hydrophobic PCL; approximately 54% of SRB was released over 7 days.

**Fig. 7:**
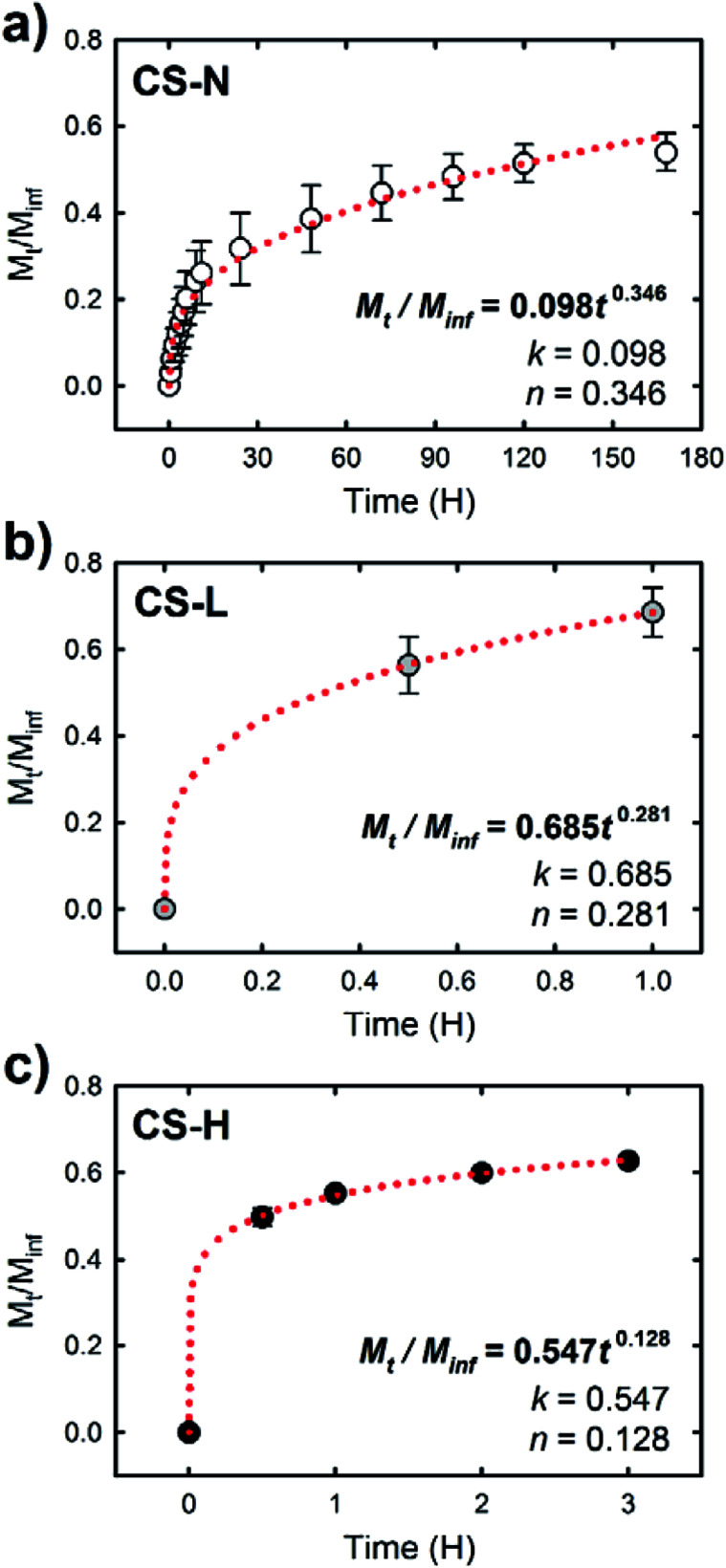
Fractional drug release, *M*_*t*_/*M*_inf_, *versus* release time for (a) CS-N, (b) CS-L, and (c) CS-H. The period of the release time was selected for *M*_*t*_/*M*_inf_ ≈ 0.6, and the red dotted lines show the fitting curves by the Ritger–Peppas model.

The release study demonstrates that the composition of fiber shell largely contributed to the release behavior of SRB in the fiber core. To characterize the release behavior of each fiber, the obtained release profiles were fitted with the Ritger–Peppas model:^40^*M*_*t*_/*M*_inf_ = *kt*^*n*^where *M*_*t*_ and *M*_inf_ are the mass of drug released at an elapsed time *t* and as time approaches infinity (*i.e.*, the total amount of the drug in the fiber mat), respectively, *k* is a constant incorporating structural and geometric characteristics of the delivery system, and *n* is the diffusional exponent, which is describing the release mechanism. The values of *n* and *k* were calculated by fitting the drug release profiles in the initial period, where *M*_t_/*M*_inf_ ≈ 0.6 (Fig. 7). The fitted exponent values of *n* were 0.346, 0.281, and 0.128 for CS-N, CS-L, and CS-H, respectively, suggesting that the Fickian diffusion is a dominant release mechanism.^40^ This indicates that the release of SRB from the core–shell fibers over the time period (*M*_t_/*M*_inf_ ≈ 0.6) was governed by molecular diffusion through the fiber shell.

Under the diffusion controlled release process, permeability of the release medium and SRB through the PCL fiber shell would determine the release rate. Along with the larger *k* values of CS-L and CS-H, the addition of water-soluble PVP into the fiber shell played a significant role in promoting mass transport rate through the fiber shell (Fig. 6). Upon exposure to the aqueous environment, dissolution of PVP domains in the shell of CS-L and CS-H would create pathways for SRB diffusion, contributing to the initial SRB release. From the WCA measurements and microphase separation imaging, it was inferred that the mass transfer of SRB would be promoted with the aid of PVP, and the molecular weight of PVP is an important parameter affecting the SRB diffusion. In fact, the SRB release was retarded in CS-H, probably due to slower swelling and dissolution of the higher molecular weight PVP compared to CS-L. In contrast to CS-L and CS-H, the fiber with PCL-only shell (CS-N) showed considerably reduced initial burst, indicating that the PCL shell served as a diffusion barrier. It is speculated that the diffusion of SRB through the defects of the shell (unintended crack or void) would be a main cause of the initial release from CS-N. The results imply that drug release profiles can be designed to some extent by a simple alteration in fiber shell formulations, such as the addition of porogens with distinct physicochemical characteristics.

### HEK viability and growth on core–shell fibers

**Fig. 8:**
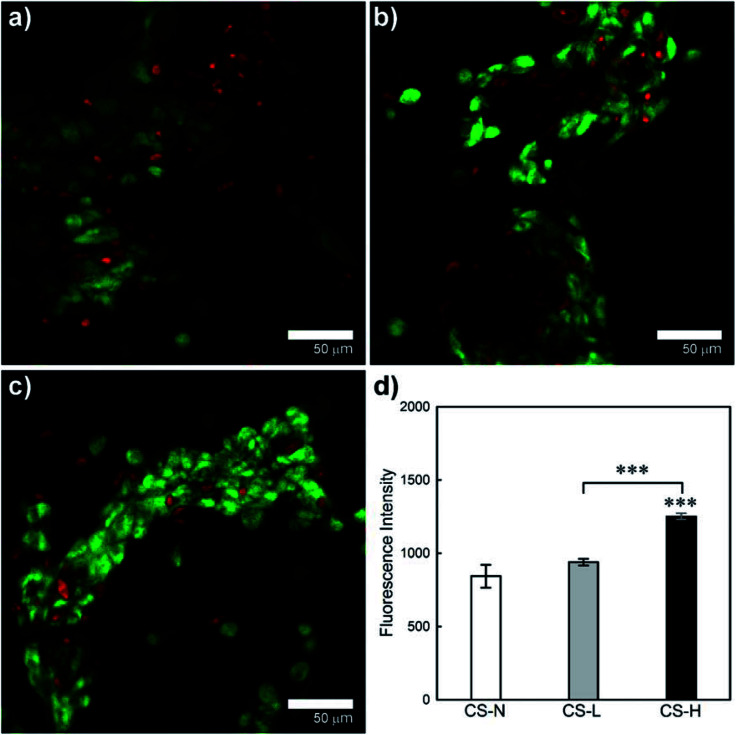
Growth of HEK cells on fiber mats with (a) monolithic PCL shell (CS-N), (b) PCL shell containing 40 kDa PVP porogens (CS-L) and (c) PCL shell containing 1300 kDa PVP porogens (CS-H). (d) Comparison of HEK proliferation at 48 h incubation. Data represent mean (*n* = 3) ± S.D. ***p < 0.001.

Viability and growth behavior of HEK cells on the solution blown core–shell fiber mats were characterized by confocal microscopy and the alamarBlue assay (Fig. 8). HEK cells were used for the cell study considering potential applications of the fiber webs in skin regeneration and wound healing. Live and dead cells in confocal images are indicated by FDA (green) and PI (red) staining, respectively. The CS-L and CS-H samples exhibited greater packing densities of viable cells with less dead cells compared to CS-N, indicating that the PVP/PCL blend shells provided a favorable environment for HEK cell proliferation.

Proliferation of HEK cells on the fiber mats was quantitatively examined by the alamarBlue assay (Fig. 8(d)). From the ANOVA and pair-wise comparison, CS-H showed significantly higher HEK proliferation than the other two samples (*p* < 0.005), while CS-N and CS-L did not show statistical differences in fluorescence intensity. The result confirms that CS-H holds the highest HEK viability, supporting the microscopic observations shown in Fig. 8(a)–(c).

**Fig. 9:**
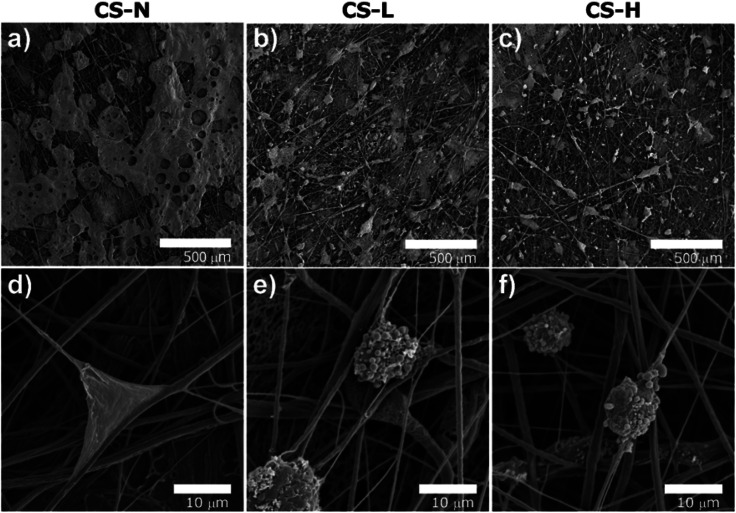
SEM images of HEK cells cultured on fiber mats with (a and d) monolithic PCL shell (CS-N), (b and e) PCL shell containing 40 kDa PVP porogens (CS-L), and (c and f) PCL shell containing 1300 kDa PVP porogens (CS-H).

The HEK cell morphology and their packing geometry on the fiber mats were assessed by SEM images (Fig. 9). The cells on CS-N formed two-dimensional monolayers (Fig. 9(a and d)), whereas the cells formed spherical clusters on CS-L (Fig. 9(b and e)) and CS-H (Fig. 9(c and f)). Three-dimensional (3D) cell construction with the spherical morphology indicates that cells proliferate while maintaining their native functions.^41^ The 3D growth of cells was more obvious in CS-H, which coincides with the quantitative data in Fig. 8(d). The result indicates that the addition of PVP (1300 kDa) created an environment favorable for HEK cells to attach, migrate, and proliferate like the native ECM.

Previous studies reported that the cell growth and proliferation on PCL surfaces could be enhanced by optimizing the surface wettability, such as through introduction of hydrophilic polymers or plasma treatment.^37–39^ If it is assumed that the PVP domains provide suitable environment for cell growth as reported in Cho *et al.*'s study,^39^ the surface with larger domains of PVP (in CS-H) may be beneficial to cell proliferation. While 40 kDa PVP dissolves rapidly in the medium, 1300 kDa PVP domains would remain longer due to the slower relaxation of high molecular weight PVP, and this may lead to a preferable environment for cell proliferation.

Another possibility comes from the creation of uneven and larger PVP domains in CS-H than CS-L. Prior researches reported that submicron-scale roughness enhanced cell adhesion and attachment by the increased surface area,^42,43^ while the optimal scale of roughness could vary depending on the cell types.^42–46^ In our study, the roughened topography of CS-H (with larger sized emulsions) would provide the most acceptable environment for cell viability and 3D proliferation. CS-H appears to be well suited for long-term culture of HEK spheroids with minimal necrosis/apoptosis, and such fiber formulations can be applied to skin regeneration and wound repair.

## Conclusion

The core–shell fibers with different shell compositions were fabricated *via* solution blow spinning. The SRB dye was incorporated in the core compartment of fibers, and its release in the aqueous medium was examined. The fibers with monolithic PCL shell displayed the reduced burst effect followed by long-term sustained release compared to PVP/PCL shell fibers. Addition of water-soluble PVP into the fiber shell accelerated drug release. Among the tested, CS-L showed the highest release rate due to the rapid dissolution of smaller PVP molecules; the cumulative release was ∼56% in 30 min and ∼94% in 7 days. The results suggest that the release rate and the burst effect can be controlled by the structural manipulation of drug-carriers, such as creation of potential diffusion pathways (PVP porogens), and by the compositional adjustment of polymer matrix. Blending PVP in the PCL shell improved the HEK cell viability and spheroid proliferations by the increased surface wettability and the roughened surface topology. The findings of this study provide simple yet fundamental guidance on the fiber formulations for controlling drug release rates and promoting cell proliferations, which would be useful for designing applications in wound healing and tissue regeneration.

## Conflicts of interest

There are no conflicts of interest to declare.

## Supplementary Material

RA-008-C8RA05485A-s001
