# Supplementary material for: Influence of shell compositions of solution blown PVP/PCL core–shell fibers on drug release and cell growth
Source: RSC Adv. 2018 Sep 19;8(57):32470–80. doi: 10.1039/c8ra05485a (PMC9086270; doi:10.1039/c8ra05485a)
Supplement: RA-008-C8RA05485A-s001 [file RA-008-C8RA05485A-s001.pdf]

Electronic Supplementary Material (ESI) for RSC Advances.
This journal is © The Royal Society of Chemistry 2018

Supporting Information

Influence of Shell Compositions of Solution Blown PVP/PCL Core-Shell Fibers on Drug Release and Cell Growth

Seok Chan Park[†], Min Jung Kim[†], Kyoungju Choi[†‡], Jooyoun Kim[§^], and Seong-O Choi*[†‡]*

[†]Nanotechnology Innovation Center of Kansas State (NICKS), Kansas State University, Manhattan, KS, USA

[‡]Department of Anatomy and Physiology, College of Veterinary Medicine, Kansas State University, Manhattan, KS, USA

[§]Department of Textiles, Merchandising and Fashion Design, College of Human Ecology, Seoul National University, Seoul, Republic of Korea

[^]Research Institute of Human Ecology, Seoul National University, Seoul, Republic of Korea

*E-mail: jkim256@snu.ac.kr (J. K.), *E-mail: sochoi@ksu.edu (S.-O. C.).

SEM images of solution blown fibers with and without poly-L-lysine coating, and the EDS mapping results for CS-L and CS-H are shown along with the chemical structures of the polymers used for fiber fabrication.

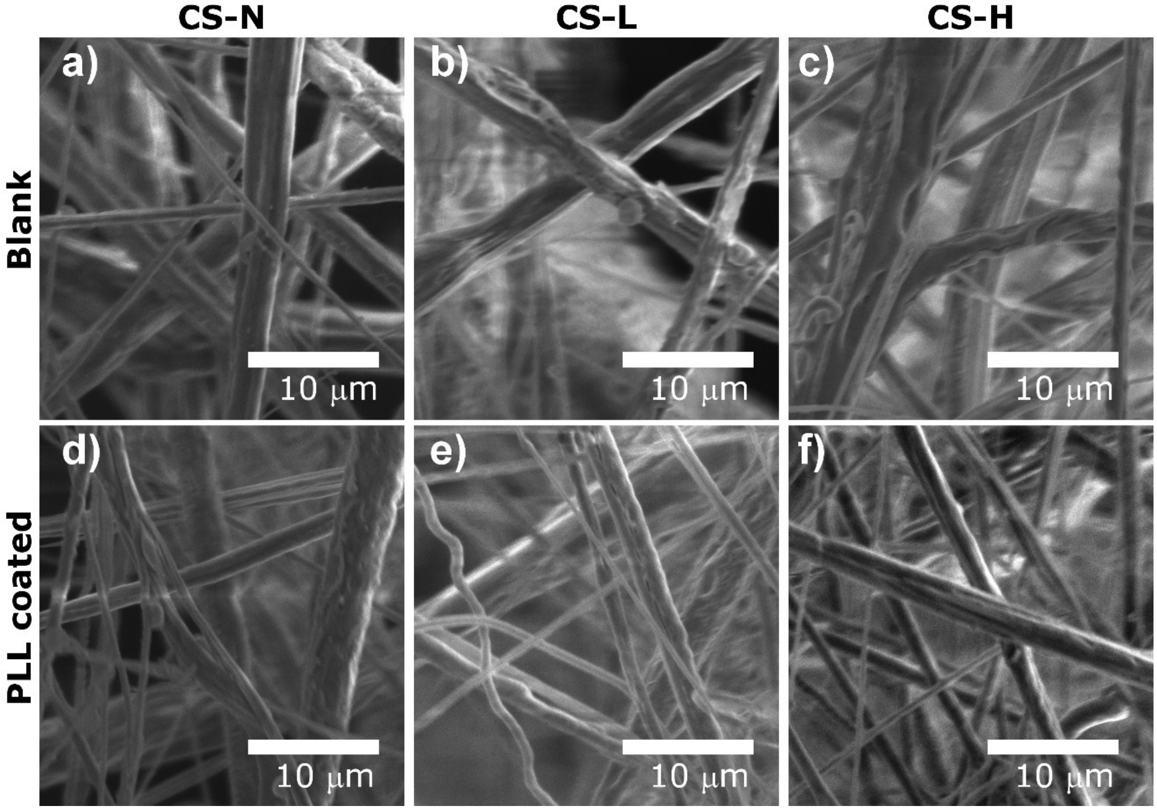

Figure S1. SEM images of solution blown fibers with (a, d) monolithic PCL shell (CS-N), (b, e) PCL shell containing 40 kDa PVP porogens (CS-L), and (c, f) PCL shell containing 1300 kDa PVP porogens (CS-H). The blank fibers (a, b, c) were immersed in DI water for 3 h, while (d, e, f) poly-L-lysine coated fibers were immersed in poly-L-lysine solution for 3 h.

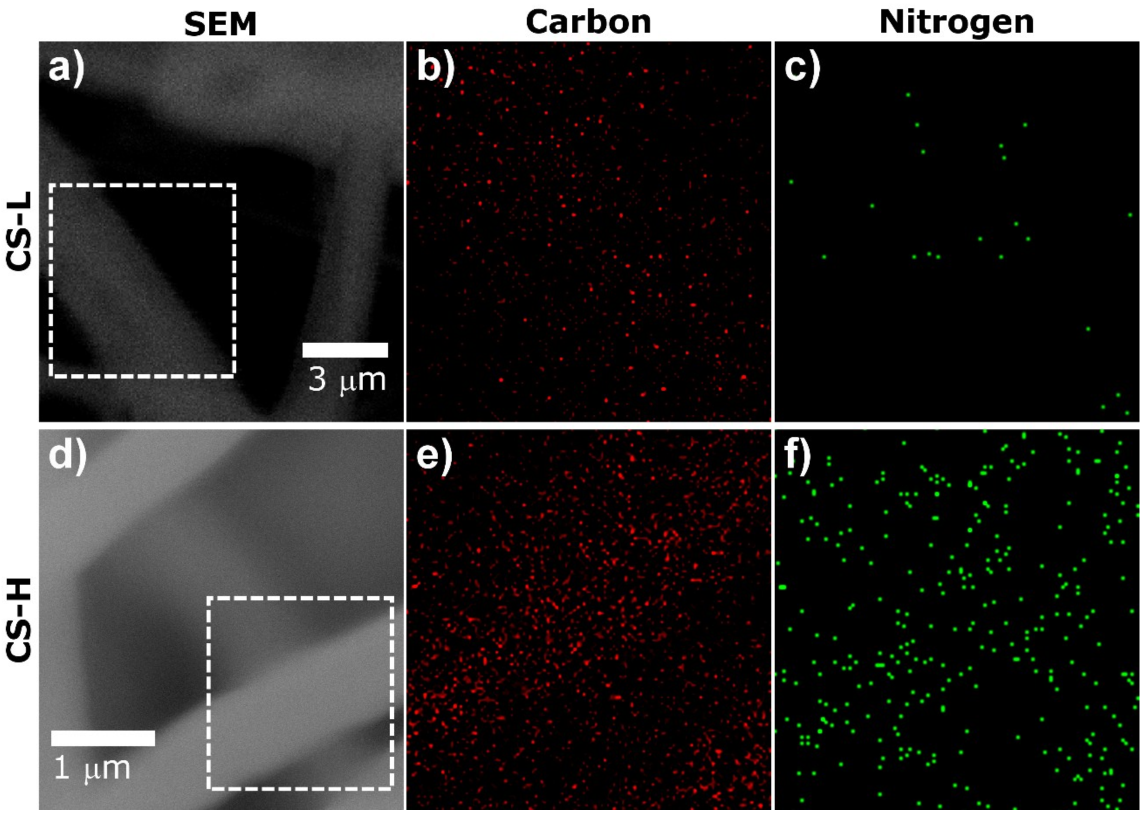

Figure S2. SEM images and elemental maps of core-shell fibers with (a, b, c) PCL shell containing 40 kDa PVP porogens (CS-L) and (d, e, f) PCL shell containing 1300 kDa PVP porogens (CS-H). Red spots in the elemental maps (b, e) represent the distribution of carbon, and green spots in the elemental maps (c, f) exhibit the nitrogen distribution on the fibers.

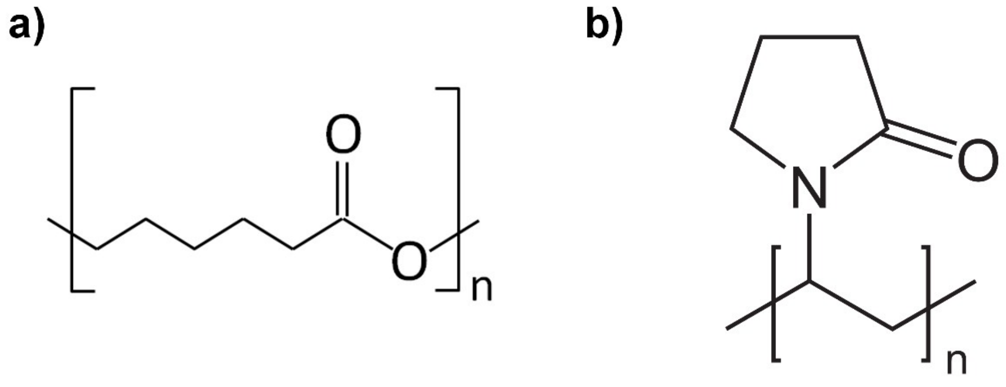

Figure S3. Chemical structure of (a) PCL and (b) PVP polymers used for fiber fabrication.
